# Do Intergroup Conflicts Necessarily Result from Outgroup Hate?

**DOI:** 10.1371/journal.pone.0097848

**Published:** 2014-06-04

**Authors:** Michael Mäs, Jacob Dijkstra

**Affiliations:** 1 ETH Zurich, Chair of Sociology, in particular of Modeling and Simulation, Zurich, Switzerland; 2 Department of Sociology/ICS, University of Groningen, Groningen, The Netherlands; University of Maribor, Slovenia

## Abstract

We developed a new experimental design to test whether or not individuals engage in conflict between social groups because they seek to harm outgroup members. Challenging prominent social psychological theories, we did not find support for such negative social preferences. Nevertheless, subjects heavily engaged in group conflict. Results support the argument that processes that act within social groups motivate engagement in conflict between groups even in the absence of negative social preferences. In particular, we found that “cheap talk” communication between group members fuels conflict. Analyses did not support the notion that the effect of communication results from guilt-aversion processes.

## Introduction

Contributing to the production of public goods is collectively efficient but it is not individually rational. Scholars from various academic disciplines are therefore puzzled by the high contributions to the production of collective goods that have been observed in field research [1]–[3] as well as laboratory experiments [4], [5]. Even more puzzling, however, are the high contribution rates that have been found in intergroup-conflict games [6]–[9], a setting where contributions are neither individually rational nor collectively efficient. In this paper, we report results of two experiments that were designed to test why individuals engage in intergroup conflict.

In an intergroup-conflict setting, the population consists of distinct social groups, each of which faces an intragroup collective good problem. In addition, contributions to the collective good of one’s group create negative externalities for the other group in that they reduce the value of the collective good of the other group. Typical examples of intergroup-conflict settings range from team sports such as soccer, to TV talent shows where fans vote for contestants, to competition for market share between organizations, to election campaigns in which political parties compete for voters, to violent conflicts between nations. In each of these cases, each individual (e.g., each player of a soccer team or each member of a political party) has to decide how much to contribute to the public good of her own group. Contributions produce benefit for all group members but also entail considerable costs for the contributor, rendering contribution individually irrational and creating the intragroup collective-good problem. In addition, contributions to the group’s public good decrease the welfare of others outside the own group: the chances of winning for the competing sports team become slimmer; the market shares of other organizations decrease; the number of votes for the competing political parties decreases; the number of enemy casualties of war increases.

Obviously, rational egoists will not contribute to the collective good of their group and, therefore, will not engage in intergroup conflict. Nevertheless, everyday experience as well as experimental research [6], [7] demonstrates that populations composed of several distinct groups can end up in very inefficient situations where members of different subgroups contribute to the collective good of their group but where these contributions harm outgroup members to such an extent that all individuals are worse off than if nobody had contributed in the first place.

Scholars debate which individual motives underlie contributions in intergroup conflict situations. On the one hand, social-identity theory [10] assumes that individuals seek to maximize status differences between salient in- and outgroups [11], [12]. Supporting this notion, experimental research along the lines of the minimal-group paradigm found that subjects prefer money allocations that maximize payoff differences between groups even if this decreases their individual payoff [13]. On the other hand, classical theories of intergroup prejudice [15] and more recent theories of intergroup categorization [16] hold that group membership implies negative feelings towards a salient outgroup only under very limited conditions. Instead, these approaches hold that not “outgroup hate” but “ingroup love” motivates contributions to intergroup conflict. In other words, this view holds that individuals may engage in conflict that implies substantive negative externalities for outgroup members mainly because they seek to support the welfare of their fellow group members. In this view, the fact that contributions to conflict harm the outgroup is a mere (perhaps unfortunate) by-product.

Empirically identifying the motives that drive contributions to a given intergroup conflict is an intricate problem [7], [8], as high contributions may result from positive social preferences towards members of one’s group, but may also be motivated by the desire to harm members of the other group. Similarly, low contributions may reflect purely selfish motives or an irenic seeking to avoid harming the other group.

There is little empirical research on the motives that underlie contributions to a given intergroup conflict. Halevy et al. [8] presented an experimental design in which subjects who decided to contribute to the collective good of their group had two options. Either they contributed in a way that would not affect the payoffs of outgroup members or in a way that would also decrease the payoffs of the other group. It turned out that contributors hardly chose the option that harmed outgroup members, providing first support for the claim that subjects did not intend to decrease payoffs of outgroup members. In a more elaborate experimental design Abbink et al. [6] found contributions to the group public good to be substantially above the Nash equilibrium prediction (which was strictly positive in their design). Furthermore, the authors reported that there were no significant effects of outgroup past behavior on individuals’ contributions and conjectured that “subjects seem to focus more on the interaction with the other team members than on that with the rival team, but at this point one can only speculate whether this can be generalized and how this is best explained” [6].

Further doubt on the outgroup-hate mechanism was cast by recent psychological research [17] on the effects of oxytocin, a peptide that is produced in the hypothalamus. It turned out that subjects given oxytocin show increased ethnocentrism mainly because of increased ingroup favoritism and to a much lesser extent because of increased outgroup derogation.

Inspired by these empirical findings, we study in this contribution whether individual engagement in intergroup conflict is motivated by negative social preferences towards the outgroup (outgroup hate). To this end, we developed a simple formal model of intergroup conflict that takes into account social preferences towards in- and outgroup members. Analyzing the model, we developed a new experimental design which allows drawing conclusions about the nature of other-regarding preferences. Results of two laboratory experiments that apply this experimental design show that, in the setting of our experiment, contributions to intergroup conflict were not motivated by outgroup hate, a finding that challenges psychological theories of intergroup relations [10], [11].

The absence of outgroup hate, however, does not exclude that individuals heavily engage in conflict with the other group, decreasing their own as well as collective welfare. In contrast, as conjectured by Abbink et al. [6], individuals may contribute to conflict between groups as a result of a social process that acts within their group even if they do not have negative social preferences. This is a striking conjecture as it suggests that conflicts between groups that we observe in real life may not always be caused by negative intergroup relations but may, instead, be the result of seemingly innocent social processes that act within the groups.

In order to test this conjecture, we studied in the second experiment whether intergroup conflict is fueled when we allow subgroup members to engage in cheap-talk communication, a social process that should not affect decisions by rational egoists but that has been shown to increase contributions in public-good experiments [7], [18]–[20]. Using the new experimental design, we did not find outgroup hate in the second experiment. Nevertheless, subjects contributed on average 89 percent of their endowment to intergroup conflict when they could send short messages to their group members, an increase of 31 percent compared to the condition without communication.

In addition, the second experiment was specifically designed to test why communication increases contributions, a critical question because communication comprises a multitude of social processes [5] that need to be carefully disentangled in order to understand communication effects. Recent economic models [21], [23], [24] suggest that the statement of intentions during communication increases contributions if subjects seek to avoid guilt, the psychological costs that they perceive if they let others down who were influenced by their stated intentions. Our results do not support that this mechanism underlies the very strong increase in contributions that results from communication. Instead, explorative analyses suggest that communication increases intergroup conflict in our experiment because subjects responded to positive reinforcement by their fellow group members. This resembles the mechanism Coleman [26] invokes to explain the occurrence of ‘excessive zeal’ in the production of public goods. Expressions of encouragement and gratitude (i.e., positive reinforcement) are dispensed at low costs but may be highly valued by the receiver, spurring him on to yet greater contributions.

## Experimental Method

The main purpose of this study was to test whether intergroup settings lead to positive or negative social preferences regarding outgroup members and whether high engagement in intergroup conflict is possible even in the absence of outgroup hate. To this end, we present in the following a game-theoretic model of intergroup settings that takes into account social preferences regarding members of both individuals’ ingroups and outgroups. Based on this model, we identified two experimental conditions which can be compared in order to draw conclusions about the nature of preferences regarding outgroup members.

Two central features characterize intergroup settings. First, individuals are members of one of two distinct social groups of 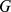 members. Members of each group face an intragroup public-good problem. In our model of intergroup settings, players simultaneously decide how many money units 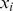, of an initial endowment of 10 money units, to contribute to the group public good. Player 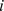
’s material payoff 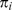 as a function of ingroup contributions is
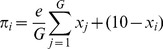
(1)where 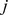 identifies the 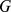 ingroup members only. In our experimental studies, we focused on groups of three actors (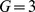) and assigned the value two to the efficiency factor 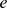 (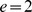) in order to simplify calculations for subjects.

Second, in intergroup settings individual contributions to one’s group’s public good also affect the material value of the public good of the respective outgroup to a degree that is described by parameter 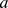. This intergroup relationship is reciprocal. Player 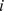
’s material payoff 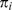 as a function of ingroup (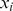) and outgroup (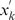) contributions is then summarized in Equation 2, where 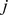 again identifies the ingroup members and 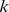 identifies the outgroup members.
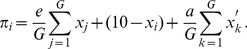
(2)


**Figure 1:**
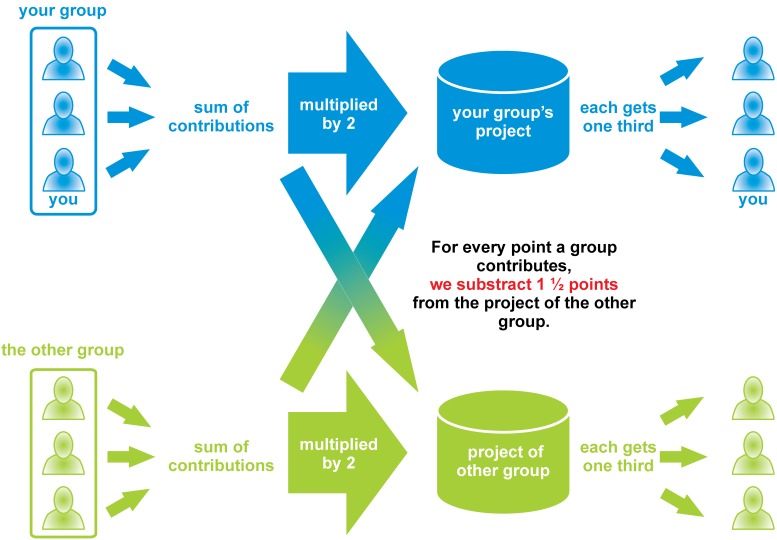
Illustration of the Game Structure in the Conflict condition that was used in the experiments.

If parameter 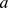 adopts the value zero, then each group separately plays a standard public good game (standard PGG). In the experiments, we compare contributions under two conditions. In the first condition, denoted “Conflict”, parameter 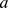 adopts the value −1.5. This implements that a contribution of one money unit to the public good of one’s group decreases the value of the public good of the respective other group by 1.5 money units. This value of 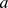 implies that there is global inefficiency when all subjects contribute at the same level in Conflict: through the losses imposed by the other group, all members of both groups are worse off than if they had kept their endowment of 10 money units and contributed nothing. Figure 1 illustrates the game structure of the Conflict condition. This figure was also used in the instructions of the experiments.

The second condition is characterized by 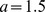 and will be referred to as “Harmony”. Under this condition, each contributed money unit implies an increase in the value of the outgroup’s public good by 1.5 money units. Note that since the outgroup consists of 3 players, the impact on each individual outgroup member is 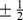. In the remainder of this section, we demonstrate that a comparison of contributions in these two conditions allows conclusions about individual preferences towards outgroup members.

Modeling individual contribution decisions, we assumed a simple linear ‘altruism model’ where the altruism parameters may adopt different values for members of different groups and are allowed to be negative. To be more precise, individual 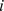
’s utility is linear in money, and is a weighted sum of own material payoffs and the material payoffs that in- and outgroup members (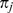 and 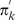) receive:
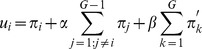
(3)


A player’s utility function thus has two social preference parameters 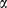 and 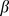. Parameter 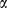 represents the evaluation of payoffs that members of one’s ingroup receive and 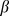 models the evaluation of outgroup members’ payoffs. Positive parameter values imply that individuals derive a positive utility from each money unit that in- or outgroup members earn, incorporating the psychological concept of “ingroup love”[16]. Negative values, on the other hand, imply that individuals are unhappy if members of the respective group make profit. This corresponds to what is referred to as “outgroup hate” in psychology [16]. We presume that players exhibit (weak) ingroup favoritism in the sense that they value payoffs of ingroup members at least as highly as outgroup payoffs (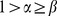). In addition, since 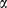 and 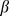 are both below 1, players are assumed to be selfish to some extent: giving a dollar to someone else strictly decreases their utility.

In the following, we first focus on model predictions for a one-shot stage game under Harmony and Conflict. Subsequently, we turn to infinitely repeated games and compare the ‘trigger strategy’ Nash equilibria as functions of the players’ social preferences for ingroup and outgroup members.

### The Stage Game

Given the game and the assumptions outlined above, we can analyze the one-shot stage game. In Harmony, the marginal utility of contributing to the group’s public good is 
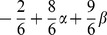
. In Conflict, the marginal utility of contributing is 
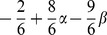
. It is of course possible to make contributing individually rational by making 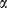 and 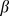 large enough. However, this would amount to simply assuming away the problem of cooperation between rational individuals. If 
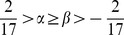
, contributing nothing in the stage game is the unique dominant strategy in both Harmony and Conflict. In the following we will therefore assume that the parameters are bounded in this way, which gives us a unique equilibrium in the stage game where every player contributes nothing and earns a material payoff of 10. For each player 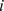 the associated utility is:
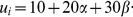
(4)


### The Infinitely Repeated Game

Repeating the stage game indefinitely allows for the existence of Nash equilibria other than the ‘no contribution’ strategy profile in the one-shot stage game. Throughout we assume that players discount future payoffs by a constant discount parameter 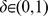 and focus on two different symmetric trigger strategy profiles [25]. We look for symmetric strategies since in the experiment the subjects have perfectly symmetrical roles. Obviously, there can be many asymmetric equilibria, but since all subjects occupy an equivalent position, there is no a priori reason to assume that an asymmetric equilibrium will be played. We focus on trigger strategy equilibria first of all because they are subgame perfect, given that not contributing anything is an equilibrium in the stage game. Secondly, in a mathematically tractable and stylized way the trigger strategies embody the notion that a player might attempt high levels of contributions in the beginning of the game, after which she decreases her contributions because of disappointing contributions by others.

First consider the contribution profile where all players across both groups contribute a nonzero part of the endowment (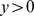), and the following symmetric (trigger) strategy profile: “Contribute 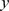 in every round as long as no player has deviated (Contribution Phase). When a single player has deviated, contribute nothing forever after (Punishment Phase).” We investigate whether this strategy profile can be an equilibrium.

The utilities in each round of the Contribution Phase are
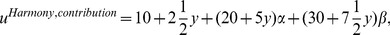
(5)and
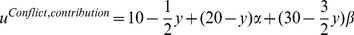
(6)in Harmony and Conflict, respectively. In both Harmony and Conflict the utilities in each round of the Punishment Phase (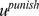) are equal to equation (4). In Conflict we now have 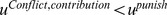 for any 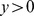. Thus, a symmetric contribution profile across both groups, with strictly positive contributions cannot be an equilibrium under these trigger strategies in Conflict. However, numerous experimental studies found substantial contributions in intergroup-conflict settings [6], [7]. Therefore, we look for a symmetric equilibrium trigger strategy profile that allows for strictly positive contributions in Conflict.

Consider the contribution profile where all players who belong to the ingroup contribute 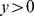, and the following ingroup symmetric trigger strategy profile: ‘Contribute 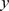 in every round as long as no ingroup member has deviated (Contribution Phase). When a single ingroup member has deviated, contribute nothing forever after (Punishment Phase).’ According to this profile, although players still care about the payoffs of outgroup members (i.e., we have made no changes in the utilities, but only in the strategies), their strategies are based on behavior within their own groups only: only deviations by ingroup members are punished. Note that this strategy profile chimes with the previously cited observation of Abbink [6] that subjects focus more on the interaction with fellow team members than on interactions with the rival team. In addition, the data from the experiment allows us to evaluate the validity of this assumption (see section II.C). In the following, we investigate whether this strategy profile can be an equilibrium.

Let 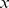 denote the average contribution in the other group. Then a player’s utility in each round of the Contribution Phase in Harmony is
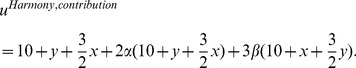
(7)


The maximum utility a player can get in a single round by deviating is obtained when she contributes nothing to the group public good, while her fellow ingroup members continue contributing 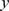. The associated ‘temptation payoff’ in Harmony is
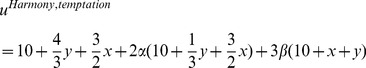
(8)Since the punishment takes place only within the group we finally have
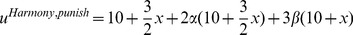
(9)It is easy to show that the ingroup symmetric trigger strategy profile is a Nash equilibrium of the infinitely repeated game whenever the discount parameter satisfies
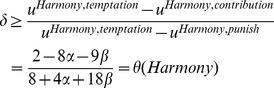
(10)
Equation 10 shows that the equilibrium threshold (i) is independent of both y and 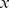, and (ii) decreases in both 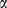 and 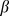.

Performing the same analysis for Conflict, we find that the utility in each round of the Contribution Phase is
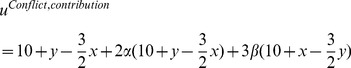
(11)The temptation and punishment payoffs are
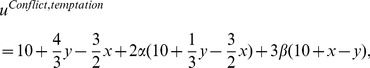
(12)and
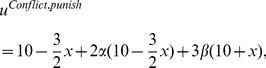
(13)respectively.

By 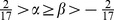, conforming to the ingroup symmetric trigger strategy profile is better than being punished, in Conflict. The ingroup trigger strategies form an equilibrium in Conflict whenever the discount parameter satisfies
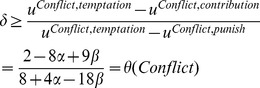
(14)
Equation 14 shows that the equilibrium threshold (i) is independent of both 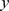 and 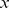, (ii) decreases in 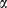, and (iii) increases in 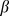.

The equilibrium analysis of the ingroup symmetric trigger strategy profile thus yields two threshold values for the discount parameter, 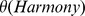 and 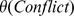. Comparing equations 10 and 14 shows that 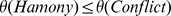 for any pair 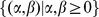. In fact, it is easy to show that 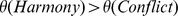 if and only if 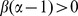. By 
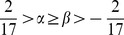
, this is true only if 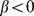.

Summarizing the theoretical results for our experimental games, we have found an ingroup symmetric strategy profile that allows strictly positive contributions in both Harmony and Conflict, while not contributing anything is the unique dominant strategy in the one-shot stage game. In addition, under the current model and equilibrium profile we can formulate the following corollary. This conclusion is valid even if ingroup social preferences (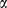) are negative, as long as 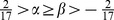.

**Corollary:** *The discount parameter threshold is higher in Harmony than in Conflict if and only if players have strictly negative social preferences for the material payoff of the outgroup. Therefore, if we observe in the experiment higher average contributions in the Conflict condition than in the Harmony condition, we can conclude that participants have strictly negative social preferences towards the members of the outgroup.*

In our experimental study we draw a simple random sample of participants. Therefore, there are no differences in the expected distributions of the discount parameter between the Harmony and the Conflict condition. This implies that the likelihood of any within-group contribution level 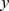 (see the equilibrium strategy profile above) being supportable in equilibrium is higher in Conflict than in Harmony if and only if 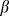 is negative; i.e., if and only if players have strictly negative social preferences for the material payoffs of the outgroup. Since the equilibrium profile does not specify a particular contribution level 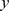, there is the problem of a multiplicity of equilibria. However, again by the fact that we draw a simple random sample of participants there are no expected differences in the particular equilibrium level 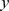, between Harmony and Conflict. Thus, observed average contributions are higher in Conflict than in Harmony if and only if participants have strictly negative social preferences for the material payoffs of the outgroup.

In behavioral terms this means the following. We first of all assume that players condition their contributions only on the contributions made by their fellow ingroup members, not on those made by outgroup members. Secondly, players are assumed to have a time preference, embodied in their discount parameter. We then showed that the critical discount parameter threshold that makes nonzero contributions sustainable in equilibrium is smaller in Harmony than in Conflict, unless players negatively value payoffs for outgroup members. In other words, any contribution level that can be sustained in equilibrium in Conflict, can be sustained in Harmony, but the reverse is not true. Thus, under the current model, contributions in Conflict will be larger than those in Harmony only in the presence of “outgroup hate”. Thus, comparing empirical contributions in Harmony and Conflict allows drawing conclusions about whether contributions are motivated by negative social preferences towards outgroup members.

Two potentially problematic aspects of the model were taken into account in the design of the experiment. First, in order to be able to apply the infinite horizon model to a laboratory setting where subjects play a fixed number of periods, we did not inform subjects about the number of periods they were playing. Second, the model is based on an ‘ingroup trigger strategy’, in which only an ingroup contribution level 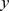 is specified in the Contribution Phase, and only ingroup deviations from this contribution level are punished. In order to be able to test to which degree subjects furthermore took into account outgroup behavior, subjects guessed contributions of their fellow ingroup members and of members of the outgroup after making their own contribution decisions. Subjects received extra payoff for guesses that were close to the actual contributions.

We make the following observation concerning the comparison of our experimental design with that of Halevy et al.[8], which has been described in the previous section. Notwithstanding the fact that the design of Halevy et al. is an excellent device to study the theoretical issue of purely negative outgroup preferences, participants’ behavior in their design is susceptible of social desirability. Making a contribution that harms the other group, when another option that benefits the ingroup equally but does not harm the outgroup is available, demonstrates negative feelings towards members of the outgroup and may be normatively very much disapproved of. Even spiteful individuals that positively hate the outgroup might refrain from this course of action because of social desirability concerns. In our current experimental design this is much less of an issue, since contributions to the group public good can easily be justified by the desire to help the ingroup. We note that this is true for the vast majority of real-life intergroup conflicts.

Comparing our design with that of Abbink et al.[6] two important differences attract attention. First of all, in their design individual contributions by egoists can be rational in the stage game, depending on the beliefs about the behaviors of others. In our design however, contributing is never egoistically rational, no matter the (expected) behaviors of others. Our design thus implies a clearer benchmark of rational egoistic behavior. Positive contributions are therefore a clear indication of social preferences. Secondly, in the Abbink et al. design teams compete for a prize, but do not positively harm each other in the competition. In other words, a team can entirely cut its losses by simply not participating in the competition for the prize. In our design, however, a group of players directly harms the other group when producing its own public good. This is a rather subtle difference that merits further experimental investigation [7].

### Ethics Statement

Both experiments were conducted at the Sociological Laboratory of the Department of Sociology at the University of Groningen in the Netherlands. Subjects were recruited from the subject pool of the Department of Sociology, which comprises mainly students and alumni from the two universities in Groningen. Volunteers registered for experimental sessions, using an online form [27]. Sessions were randomly assigned to conditions. All experiments were implemented in z-tree [28].

The recruitment and the experiment complied with the ethical guidelines set out by the Sociological Laboratory of the Department of Sociology at the University of Groningen (http://www.gmw.rug.nl/~orsee/public/privacy.php) and were approved by the Review Board of the Sociological Laboratory. Written informed consent was obtained from each participant before conducting the experiment. During the experiment subjects were made aware of the fact the experiment did not involve deception of any form.

## Experiments

### Experiment 1: Ingroup Love and Outgroup Hate

The aim of the first experiment was to empirically test whether or not negative social preferences towards outgroup members motivate contributions to intergroup conflict, applying the new experimental design. The experiment consisted of two sets of treatments. In the first set, subjects were assigned to one of two treatments, allowing a within and between subjects comparison between the Harmony and the Conflict condition. In the “first Conflict, then Harmony treatment”, subjects first made 10 decisions under the Conflict condition (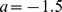) and continued with 10 decisions in the Harmony condition (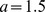). In the “first Harmony, then Conflict treatment” subjects also made 10 decisions under each condition but in the reversed order.

The second treatment set of the experiment provided two baseline conditions. In the first baseline condition, contributions to the public good of a group had no effect on the other group (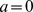). However, the remaining features of this treatment were identical to the Harmony and the Conflict condition. In particular, subjects were informed about the contributions to the public good of the other group. In the second baseline condition, subjects played a standard public good game in a group of three. In other words, subjects were not informed about contributions of another group in this condition. Below, we refer to this latter condition as the “standard public good game” (PGG). The purpose of the baseline conditions was to test whether or not differences between contributions in the Harmony and the Conflict condition might result from additional social preferences. For instance, higher average contributions in Harmony than in Conflict might result from positive social preferences towards outgroup members. In this case, one would observe higher contributions in the Harmony condition than in the baseline conditions, as subjects can increase the payoffs of outgroup members in the Harmony condition. We included the baseline conditions to test this hypothesis.

Table 1 summarizes the experimental manipulations of Experiment 1. In both baseline treatments, subjects played 20 periods under the same condition. However, since subjects in the baseline treatments never experienced a switch in the experimental conditions, we analyzed only the first 10 periods of the baseline treatments. Only these periods can be compared to the first ten periods of the first treatment set in a between-subjects design.

**Table 1:** Treatments of Experiment 1.

Treatment	Treatmentset	Condition(parameter 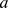 )	Information about out-groupcontributions provided	Number ofsubjects
1	1	first Conflict ( 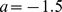 ),	yes	36
		then Harmony ( 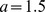 )		
2		first Harmony ( 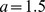 ),	yes	48
		then Conflict ( 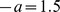 )		
3	2	20 rounds of Public	yes	48
		Good game ( 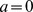 )		
4		20 rounds of Public	no	36
		Good game ( 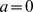 )		

#### Procedures

For each of the four treatments, we scheduled four experimental sessions with 12 subjects each. There were two societies in each session and each society consisted of two subgroups of three members (see Figure 1). Group memberships were anonymous and constant across all periods. We excluded those sessions from the analyses where too few subjects showed up to create two societies. Altogether, 36 subjects participated in the “first Conflict, then Harmony” treatment and 48 subjects participated in the treatment with the reversed order. There were 36 participants in standard PGG and 48 subjects played the PGG with information about other group 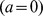.

This was the first experiment on intergroup games that had been conducted with members of this subject pool. We therefore expected that subjects did not have experiences with this type of experiment. The experiment was conducted in English language, in order to make sure that both Dutch and foreign participants had an equal understanding of the instructions.

Sessions began with general verbal instructions. Next, subjects read detailed instructions on the computer screens, receiving all instructions that concerned the public good game of their own group. In the conditions where subjects were informed about their outgroup, subjects read on the next screen that there was a second group and how this group affected their payoffs. Next, subjects interacted in 20 periods. In the first treatment set (Harmony and Conflict), subjects read after the tenth period how the rules of the game were about to be changed and then continued in the respective other condition. Subjects were not informed about the number of periods that they played in each condition.

Each interaction period consisted of four steps. First, subjects received 10 points (each worth 2 Euro cents) and decided how many points they would like to contribute. In order to assess subjects’ expectations about the contributions of their group members, subjects were asked in the second step to guess how many points the other two members of their own group had just contributed on average. In order to increase the validity of this measure [29], we informed subjects that they would receive 10 extra points at the end of the experiment for each guess that differed not more than 2 points from the real value. In the conditions where subjects were informed about an outgroup, subjects also guessed the average contribution of the outgroup members and also received 10 extra points for accurate guessing. A potential disadvantage of this method of belief elicitation is that subjects might hedge their stated guesses against the outcomes of the intergroup game [30]. We can not exclude that this was the case in the first experiment. In the second experiment, however, we refrained from including belief elicitation methods and could replicate the main findings of the first study. In the third step of an interaction period, subjects were informed about the average contributions of their group members and their outgroup (if there was one) and how many points they had just earned. Finally, subjects read whether they had earned extra points with good guessing.

Figures S1, S2, and S3 in File S1, show screenshots of the main stages of the experiment including the instructions.

#### Treatment effects

**Figure 2:**
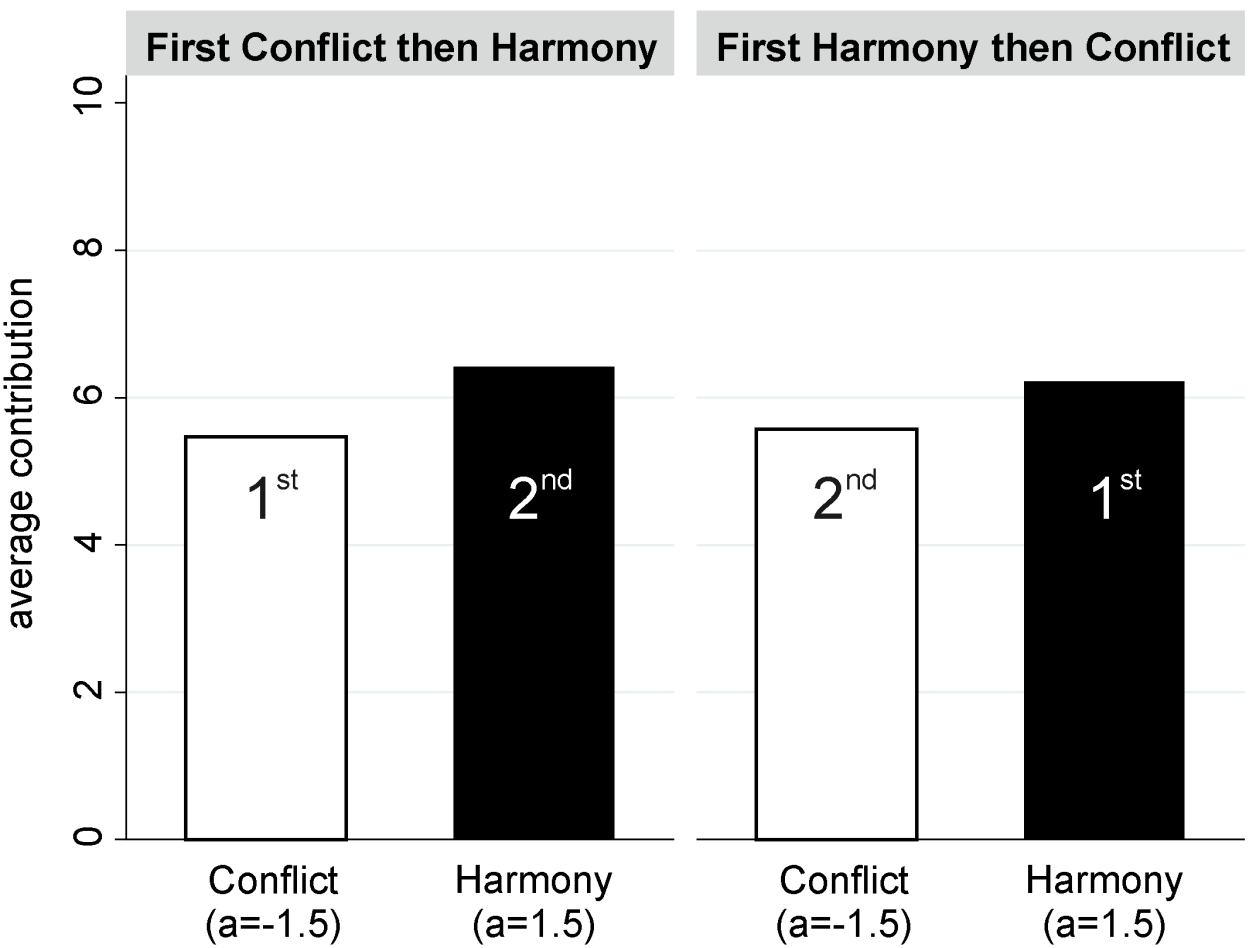
In Harmony contributions were higher than in Conflict, independent of the ordering treatment.

Figure 2 informs about the average contributions in the Harmony and Conflict condition, revealing that on average subjects contributed more points in the Harmony condition than in the Conflict condition. This was found in the “first Conflict, then Harmony treatment” and the “first Harmony, then Conflict treatment”. To test whether this difference between the two conditions was statistically significant with a method that takes into account that decisions are nested in subjects and subgroups, we estimated linear multilevel mixed-effects regression models [31], the statistical approach that we used throughout the article. Results are summarized in Table 2. A comparison of empty models with the likelihood ratio test revealed that additionally considering the nestedness in societies did not improve model fit significantly, indicating that the differences between societies were small (deviance = 2.17). The “final model” of Table 2 shows that in the Conflict condition subjects contributed on average 5.53 points. In the Harmony condition, subjects contributed 0.78 points more on average. This effect is statistically significant. Furthermore, a likelihood ratio test comparing the empty model with the final model showed that including the dummy variable for the Harmony condition significantly increased model fit (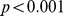). There was no significant main effect of the ordering condition (see variable “First Harmony the Conflict”). Also the interaction effect of the ordering treatment and the Harmony condition was insignificant, showing that the ordering of the Harmony and the Conflict condition did not affect contributions significantly. Likewise, likelihood ratio tests showed the fit of the model with the main effects (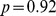) and the full model (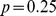) was not significantly better than the fit of the final model.

**Table 2:** Treatment effects on contributions.

	Dependent variable: Contribution			
	Empty model	Main effects	Full model	Final model
*Fixed part*				
Constant	5.92***	5. 55***	5.38***	5.53***
	(0.35)	(0.54)	(0.56)	(0.36)
Harmony		78***	95***	78***
condition		(0.13)	(0.19)	(0.13)
First Harmony		−0.04	0.11	
then Conflict		(0.71)	(0.73)	
Interaction			(0.30)	
effect			(0.26)	
*Random part*				
Between	2.87	2.87	2.87	2.87
subgroup var.	(0.94)	(0.94)	(0.94)	(0.94)
Between	1.52	1.53	1.53	1.53
subject var.	(0.35)	(0.35)	(0.35)	(0.35)
Residual	6.97	6.81	6.81	6.81
variance	(0.25)	(0.24)	(0.24)	(0.24)
−2 loglikelihood	8218.83	8181.95	8180.60	8181.95
Number of subjects	84	84	84	84
Number of decisions	1680	1680	1680	1680

Standard errors in parentheses.

***significantly different from 0 at the 1 percent level (twosided Wald-test).

**significantly different from 0 at the 5 percent level (twosided Wald-test).

*significantly different from 0 at the 10 percent level (twosided Wald-test).

**Figure 3:**
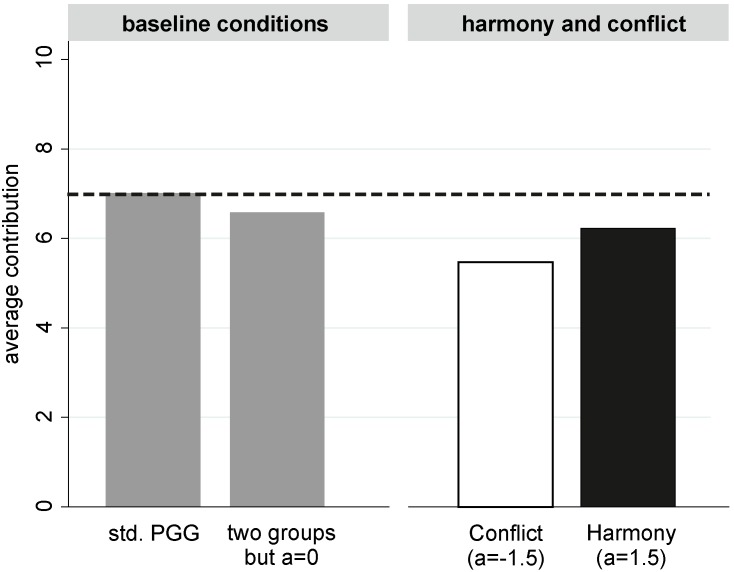
In the baseline conditions contributions were higher than in Conflict.

Figure 3 pictures differences between contributions in the two baseline conditions (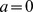) and in Harmony (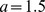) and Conflict (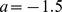). The corresponding statistical tests are reported in Table 3. This figure and the results in the table are based on the first 10 decisions. The differences between the two baseline conditions appear to be small and were not statistically significant. Furthermore, it turned out that subjects contributed on average 0.79 points less in the Harmony condition than in the standard public good game. This difference turned out to be insignificant. However, subjects in the Conflict condition contributed significantly fewer points than those subjects who played standard public good games. To be more precise, subjects contributed on average 1.55 less in the Conflict condition. A likelihood ratio test showed that fit of the full model in Table 3 is not significantly higher than the fit of the empty model (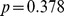).

**Table 3:** Comparison with standard public goods game.

	Dependent variable: Contribution	
	Empty model	Full model
*Fixed part*		
Constant (mean std.	6.33***	7.02***
PGG in full model)	(0.31)	(0.64)
a = 0 (PGG with		−0.44
two groups)		(0.85)
Harmony (a = 1.5)		−0.79
		(0.85)
Conflict (a = −1.5)		−1.55*
		(0.91)
*Random part*		
between subgroup	4.38	4.09
variance	(0.99)	(0.94)
between subject	2.15	2.15
variance	(0.35)	(0.35)
Residual variance	4.38	4.38
	(0.16)	(0.16)
−2 loglikelihood	7648.05	7644.96
Number of subjects	168	168
Number of decisions	1680	1680

Standard errors in parentheses.

***significantly different from 0 at the 1 percent level (twosided Wald-test).

**significantly different from 0 at the 5 percent level (twosided Wald-test).

*significantly different from 0 at the 10 percent level (twosided Wald-test).

In sum, we found that subjects contributed significantly more in the Harmony than in the Conflict condition. According to the Corollary we can, thus, conclude that Experiment 1 did not provide evidence for negative social preferences towards the members of the outgroup. Likewise, the comparison with the baseline conditions did not provide evidence for positive social preferences towards members of the outgroup.

#### Effects of the expectations

Table 4 informs about whether subjects’ contribution decisions were influenced by their expectations about the contributions of their fellow ingroup members and the members of the other group. A positive statistical effect of the expected contribution of ingroup members on subjects’ own contributions would reveal that subjects contributed more points when they expected their ingroup members to contribute many points, supporting that subjects had social preferences. Similarly, a positive effect of expected contribution of outgroup members in the Harmony condition would support that subjects sought to have similar payoffs as the members of their respective outgroup. Thus, this analysis allows us to evaluate the validity of our modeling assumption that subjects condition their own contributions on the contributions of their ingroup only.

**Table 4:** Effects of expected ingroup and outgroup contributions.

	Dependent variable: Contribution		
	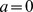	Harmony( 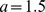 )	Conflict( 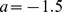 )
*Fixed part*			
Constant	0.99**	0.96**	0.06
	(0.50)	(0.45)	(0.52)
Expected contribution	0.80***	0.84***	0.89***
ingroup members	(0.06)	(0.07)	(0.07)
Expected contribution	0.08	0.03	0.08
outgroup members	(0.05)	(0.07)	(0.08)
*Random part*			
between subject	2.80	2.96	1.53
variance	(0.62)	(0.66)	(0.35)
Residual variance	2.29	2.70	6.81
	(0.16)	(0.18)	(0.24)
−2 loglikelihood	1883.59	1957.44	1620.05
Number of subjects	48	48	36
Number of decisions	480	480	360

Standard errors in parentheses.

***significantly different from 0 at the 1 percent level (twosided Wald-test).

**significantly different from 0 at the 5 percent level (twosided Wald-test).

*significantly different from 0 at the 10 percent level (twosided Wald-test).

If, contrary to our assumption, subjects in the Conflict condition sought to increase payoff differences between their ingroup and members of the outgroup as social psychological theories suggest [10], one would expect that subjects contributed more points themselves when they expected outgroup members to contribute many points. This is because contributions of outgroup members increase the average payoff of outgroup members and, in addition, decrease the payoffs of ingroup members. Thus, if subjects sought to achieve a favorable comparison for their own group, they would respond with higher contributions to high expected contributions of outgroup members, because own contributions would increase payoffs of ingroup members and decrease payoffs of outgroup members. In the statistical model, such conditioning of contributions on the outgroup would be supported by a positive effect of expected contribution of outgroup members on subjects’ own contributions.

All analyses in Table 4 are based only on the first ten periods in the three treatments where there was an outgroup. The decision in the remaining periods were excluded because in the treatment where the two groups did not affect each other (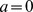) there was no change of experimental conditions after the tenth period. As a consequence only decision during the first ten periods can be compared. The table reports a separate model for each of the three conditions. Likelihood ratio tests revealed that controlling for the nestedness of decisions in subjects increased model fit significantly. However, taking into account the nestedness in subgroups and societies did not increase model fit.

We found that in all three conditions contributions were significantly influenced by the expectations about ingroup members’ contributions. In other words, subjects contributed more points when they expected their group members to contribute many points. In a model (not shown here) of the data from all three conditions we did not find significant differences in the effect size of ingroup expectations.

Importantly, this effect was not found for the expectations concerning the outgroup. In neither of the three conditions, did subjects contribute more or less depending on their expectations concerning the contributions made by the outgroup. This is most surprising for the Harmony condition, because contributions of both in- and outgroup members increased subjects’ own payoffs. Nevertheless, categorizing subjects into two groups appeared to have affected subjects’ decisions in the sense that they paid attention only to ingroup contributions when making their own contribution decisions [12]. This supports the modeling assumption we made and contradicts the social psychological notion that subjects seek to increase payoff differences between their ingroup and members of the outgroup.

In a nutshell, we found contributions in the Harmony condition to be significantly higher than in the Conflict condition. Furthermore, contributions in both Harmony and Conflict turned out to be lower than in the baseline conditions, suggesting that the difference between contributions in Harmony and Conflict were not caused by additional incentives to contribute in Harmony. Finally, we found support for our assumption that subjects based their own decisions on expected contributions by ingroup members only. On the whole, these findings do not provide any evidence for negative social preferences towards the outgroup. In other words, in the setting of this experiment, we did not find support for the outgroup-hate argument. Furthermore, the results suggest that subjects did not seek to increase outgroup members’ payoffs. However, subjects refrained from engaging in behavior that would harm outgroup members, which explains why we found higher contributions in the Harmony conditions than in the Conflict condition.

### Experiment 2: Intragroup Processes

Results of Experiment 1 did not provide support for the outgroup-hate argument, suggesting that in the setting of this experiment subjects did not seek to increases payoff differences between members of their own group and the outgroup. Nevertheless, we found high contributions in the Conflict condition (5.53 points on average, see Table 2). The purpose of Experiment 2 was to test whether an intragroup process can increase contributions and intensify intergroup conflict. In other words, we sought to test whether or not individuals who do not seek to harm members of an outgroup may nevertheless be motivated to heavily engage in intergroup conflict as a result of a process that acts within their own group. Abbink et al.[6] provided recent evidence that this may be possible, conducting an intergroup-conflict experiment where subjects were given the opportunity to punish their ingroup members. It turned out that contributions were significantly higher than in a condition without the opportunity to punish. The experimental design of Abbink et al. does not allow to draw conclusions about the impact of outgroup hate. However, their results suggest that subjects fueled intergroup conflict not because they sought to harm the outgroup but because they sought to prevent being punished by their group members for not contributing to the collective good of their own group.

We focused in our second experiment on an alternative intragroup process, communication between group members. Many experimental studies conducted by social psychologists have demonstrated that communication has a striking potential to increase cooperation in collective-good dilemma games [19]. Recent findings from the economics literature support this finding [20]. In addition, the research group formed around Gary Bornstein [8], [32] conducted numerous intergroup-conflict experiments where subjects communicated with the members of their group before making individual decisions and found communication to increase contributions to conflict substantially.

The evidence that psychological research provided for the effects of communication on contributions is impressive. However, in the majority of communication experiments subjects met in a separate room and openly discussed the decision problem before they decided about their actual contributions. This communication process is extremely complex, creating a black box [5] that makes it very difficult to extract the core mechanisms that are responsible for the effect of communication on contributions. Identifying these core mechanisms, on the other hand, is a critical step in the development of an informative model of decision making and communication in intergroup games.

Recently, economists developed rigorous models of communication in games, focusing on the statement of intentions during communication [21], [23], [24]. According to the conventional game-theoretic framework, stating one’s intentions in an intergroup-conflict game is cheap talk [18] and should therefore fail to affect contributions. The reason is simple. Players profit from contributions of their group members independent of their own contributions. Thus, if there is only a slight chance that group members positively respond to stating high intentions, even players who do not plan to cooperate will pretend to contribute. It follows that others’ stated intentions to contribute do not inform about their actual intentions and should therefore fail to affect contribution decisions. On the other hand, empirical studies support that cheap talk increases contributions to collective goods. For instance, Duffy[33] conducted one-shot prisoner-dilemma games where subjects were either informed about the decision of their current interaction partner in the previous period or received a message from this player where she stated her intentions. Strikingly, contribution rates did not differ significantly between the two conditions but where significantly higher than in a control condition where no additional information was provided.

The effects of communicating intentions on contributions has been attributed to guilt aversion, the tendency to avoid the psychological costs of letting others down [21], [23], [24]. In a nutshell, it has been argued that individuals will stick to their stated intentions even when it is cheap talk because they would feel guilty if they contributed less than their group members expect them to contribute based on their stated intentions [22], [34]–[36]. With regard to intergroup-conflict settings, one would expect that the opportunity to inform group members about one’s intentions increases actual contributions if two assumptions are met. First, individuals tend to promise contributing more than they actually intend, e.g. in order to convince other group members to increase their contributions. Second, individuals tend to contribute in accordance with their stated intentions, because of guilt aversion.

In Experiment 2, we studied three communication conditions. First, there was a control condition without communication. Second, in the “standardized-messages condition”, subjects informed their group members about their intentions before they entered their actual decision. The purpose of including this condition was to test whether informing group members about one’s intentions and being informed about their intentions does indeed increase contributions, as the guilt-aversion argument suggests.

However, the communication of intentions is only one aspect of communication and it is questionable whether it is the crucial aspect that is responsible for the overall effect of communication on contributions. We therefore added a third condition where communication was not restricted to informing each other about intentions. However, we refrained from allowing the open face-to-face pregame discussions which many psychological experiments are based on [19]. Instead, we gave subjects the opportunity to send a short message (140 characters) to their group members. Accordingly, this condition is called the “short-message condition”. In this condition, subjects had the opportunity to state their intentions (like in the standardized-messages condition). However, they could also formulate expectations in the sense of articulating a social norm of how many points their group members should contribute. Furthermore, subjects could react to each others’ previous contributions, endorsing or criticizing their previous decisions. Finally, the transmission of short messages made it possible to communicate rather detailed arguments about why one should contribute or not.

Thus, communication in the condition with short messages was rather complex. However, the communication of short messages excludes nonverbal communication which is very difficult to measure or control experimentally [5]. Another advantage of short messages is that they can be saved, allowing us to analyze the content of the messages and explore in more detail why communication may increase contributions.

To our knowledge, there is very little research on intergroup games that involved communication and tested for negative social preferences towards outgroups [8]. We therefore conducted for each communication condition separate Harmony and Conflict treatments, applying the same method as in the first experiment. Altogether there were two (Harmony vs. Conflict) times three (no communication vs. standardized messages vs. short messages) treatments.

#### Procedures

For each of the six treatments, we conducted two sessions with 12 subjects each. In total, 144 subjects participated in this experiment. Experimental sessions were randomly assigned to the six treatments. Nineteen participants (13.2 percent) of Experiment 2 had participated earlier in Experiment 1 and were, thus, familiar with intergroup games. The other participants had never taken part in an experiment with an intergroup game before. Subjects were free to subscribe to any session in which they wanted to participate. By chance this produced a pattern in which the 19 subjects that had participated in Experiment 1 were evenly distributed over the six experimental treatments of Experiment 2.

The design of this experiment was similar to the design of the first experiment. There were, however, three main differences. First, in order to keep the duration of experimental sessions below 30 minutes, subjects made only ten decisions instead of twenty. For the same reason, subjects did not enter their expectations concerning the contributions of other participants. Finally, the experiment was conducted in Dutch language, in order to make sure that subjects could formulate and understand the short messages. In the invitation e-mails that were sent to the members of the subject pool, we made explicit that the experiment was to be conducted in Dutch.

In the treatments without communication, each interaction period consisted of two steps. First, subjects entered their contribution and, second, subjects were informed about the individual decisions of their ingroup’s members and the sum of contributions of the other group.

In the conditions with communication, interaction periods also consisted of these two steps. However, there were two additional steps at the beginning of each period. In the standardized-message conditions, subjects were first asked to send a message to their fellow group members by completing the following sentence: “I will contribute 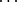 points to the group project.” Second, subjects read the messages that their respective group members had sent in the previous step. In the third step, subjects entered their actual contributions. Finally, subjects were informed about others’ decisions. In the short-message condition, subjects first entered an up to 140 characters long message and, second, read the messages of their group members. Next, subjects entered their actual contribution and were informed about the outcomes of the game.

#### Treatment effects

**Figure 4:**
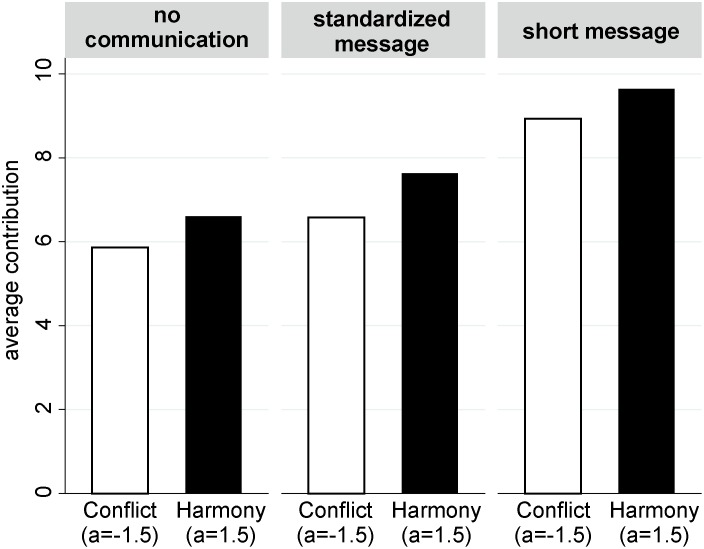
Communication increased contributions in Harmony and Conflict.

The core results of the second experiment are summarized in Figure 4, which depicts average contributions for each of the six conditions. Random-intercept models that tested the differences between conditions are reported in Table 5.

**Table 5:** Effect of communication on contributions.

	Dependent variable: Contribution		
	Empty model	Main effects	Full model
*Fixed part*			
Constant	7.54***	5.81***	5.86***
	(0.31)	(0.48)	(0.59)
Harmony		0.84*	0.75
		(0.48)	(0.83)
Standardized message		0.87	0.72
		(0.59)	(0.83)
Short message		3.05***	3.07***
		(0.59)	(0.83)
Harmony 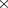 std.			0.31
message			(1.17)
Harmony 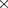 short			−0.03
message			(1.17)
*Random part*			
between subgroup	4.18	2.35	2.35
variance	(0.94)	(0.56)	(0.56)
between subject	0.58	0.58	0.58
variance	(0.17)	(0.17)	(0.17)
Residual variance	6.09	6.09	6.09
	(0.24)	(0.24)	(0.24)
−2 loglikelihood	6900. 85	6876.39	6876.29
Number of subjects	144	144	144
Number of decisions	1440	1440	1440

Standard errors in parentheses.

***significantly different from 0 at the 1 percent level (twosided Wald-test).

**significantly different from 0 at the 5 percent level (twosided Wald-test).

*significantly different from 0 at the 10 percent level (twosided Wald-test).

The first important result is that in all three communication conditions contributions were significantly higher in the Harmony condition than in the Conflict condition. On average, subjects contributed 0.84 points more in Harmony than in Conflict. The “Full model” in Table 5 shows that the differences between contributions in Harmony and Conflict did not differ between the three communication conditions (see the insignificant interaction terms). In sum, this replicates the finding of the first experiment that subjects did not have negative social preferences towards the members of the other group.

In the light of this finding, the very high contributions in the condition where subjects communicated short messages are remarkable. In the Conflict condition with short messages, subjects contributed on average 8.93 of the 10 points that they received in each period. Thus, even though there is no evidence for outgroup hate, subjects contributed most of their points and, thus, fueled the intergroup conflict.

Comparing contributions in the Conflict treatments without communication and the Conflict treatment with short-messages reveals that the very high contributions in the Conflict condition with short messages were caused by the communication. To be more precise, subjects contributed on average 3.05 points more when they could transmit short messages. This supports the claim that intergroup conflicts do not always result from negative feelings towards the outgroup but can be the result of an intragroup process.

Figure 4 shows that also in the treatments where standardized messages were transmitted, contributions where higher than in the treatments without communication. However, this difference turned out to be insignificant (see model with “Main effects” and “Full model” in Table 5). Thus, stating intentions and reading the intentions of the other subgroup members did not increase contributions significantly. This does not support the hypothesis that communication increases contributions because it increases subjects’ expectations about the contributions of the other group members. The Full model reveals that there is no indication of an interaction effect between the Harmony treatment and the communication treatments. Comparing the fit of the full model with the fit of the main-effects model showed that including the interaction effects did not improve model fit (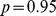). The model with the main effects, however, has a significantly better fit than the empty model (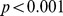).

#### Analysis of standardized messages

**Figure 5:**
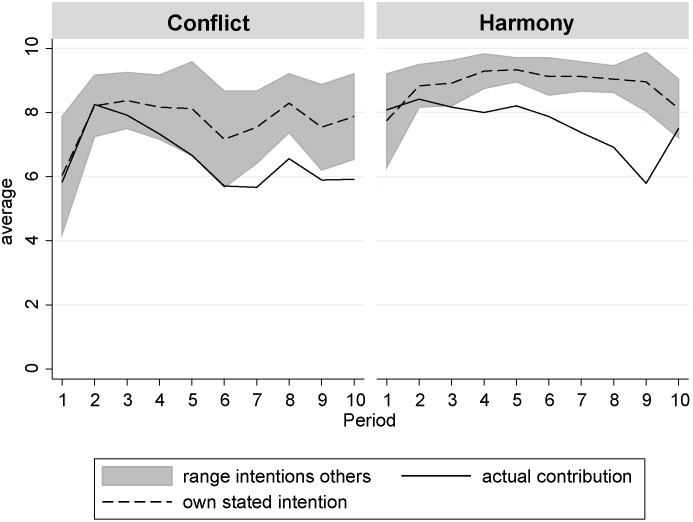
Actual contributions were lower than intentions stated in the standardized messages.

Why did the communication of intentions fail to significantly increase contributions? We did find that subjects’ decisions were correlated to their own stated intentions and those of the other group members. For the conditions where subjects transmitted standardized messages, Figure 5 shows the development of average contributions, subjects’ average intentions and the average intentions of the group members with the higher (see the upper border of the gray area) and the lower (see the lower border of the gray area) intention. The figure shows that in the Harmony as well as in the Conflict condition subjects contributed according to their stated intentions only at the very beginning of the experiment. In fact, over time average actual contributions even dropped below the average intention of the group member with the lower intention.

Table 6 informs about the degree to which contributions were influenced by subjects’ own stated intentions and the intentions of their subgroup members. First, we estimated separate random-intercept models for the Harmony and the Conflict treatment. In both models, we found significant effects of the own stated intention on actual contributions, showing that subjects did stick to their stated intentions. However, the effect sizes differ clearly between the two conditions. This is supported by the significant interaction effects between subjects’ own stated intention and the condition dummy in for instance the “Final model”.

**Table 6:** Effects of intentions on contributions.

	Dependent variable: Contribution					
	OnlyHarmony		OnlyConflict	Maineffects	Main andinteractions	Finalmodel
*Fixed part*						
Constant	5.13***	0.01		0.88	−0.07	0.38
	(1.90)	(0.94)		(0.96)	(1.15)	(0.70)
Own intention	0.27***	0.78***		0.60***	0.76***	0.76***
	(0.10)	(0.05)		(0.05)	(0.05)	(0.06)
Higher intention	0.00	0.04		0.11	0.06	
group members	(0.18)	(0.11)		(0.10)	(0.12)	
Lower intention	0.16	0.23***		0.19***	0.23***	0.23***
group members	(0.10)	(0.05)		(0.05)	(0.06)	(0. 04)
Period effect	−0.21***	−0.23***		−0.22***	−0.23***	−0.22***
	(0.06)	(0.05)		(0.04)	(0.05)	(0.04)
Harmony				0.00	4.68**	4.56***
condition				(0.50)	(2.07)	(1.06)
Own intention					−0.52***	−0.53***
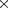 Harmony					(0.11)	(0.10)
Higher intention					−0.01	
group member					(0.21)	
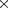 Harmony						
Lower intention					−0.03	
group member					(0.11)	
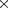 Harmony						
Period 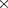 Harmony					0.02	
					(0.07)	
*Random part*						
betw. subgroup	2.22	0.01		0.71	1.04	0.99
variance	(1.25)	(0.28)		(0.36)	(0.51)	(0.49)
between subject	0.20	0.93		0.26	0.59	0.61
variance	(0.30)	(0.48)		(0.21)	(0.28)	(0.28)
Residual	6.16	4.26		5.67	5.20	5.21
variance	(0.59)	(0.41)		(0.39)	(0.35)	(0.35)
−2 loglikelihood	1141.96	1056.86		2233.27	2211.65	2212.10
Number of subjects	24	24		48	48	48
Number of decisions	240	240		480	480	480

Standard errors in parentheses.

***significantly different from 0 at the 1 percent level (twosided Wald-test).

**significantly different from 0 at the 5 percent level (twosided Wald-test).

*significantly different from 0 at the 10 percent level (twosided Wald-test).

In other words, subjects’ stated intentions were more reliable in the Conflict than in Harmony condition. It remains an open question what mechanism caused this effect. On the one hand, it could be that in the Conflict treatment subjects took the statement of their intentions more seriously, and thus adjusted their final decisions less than subjects in the Harmony treatment. Alternatively, subjects who were assigned to the Conflict conditions might have felt more morally obliged to stick to their stated intention when they made their decision [37].

Subjects were also influenced by the standardized messages that they received from their group members. However, it turned out that in both conditions subjects hardly considered the message that contained the higher intention and were mainly influenced by the lower intention. The former is demonstrated by the very weak and insignificant effects of the stated intention of the group member with the higher intention in the two separate models for Harmony and Conflict in Table 6. The latter is supported by the stronger and significant effects of the lower intention in the same models. The effect of the lower intention was not significant in the Harmony condition. However, in a model that does not include the very weak and insignificant effect of the higher intention (not reported), this effect is significant (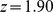). In addition, the model of Table 6 that includes interaction effects shows that the effect of the lower intention of the two group members does not differ significantly between Harmony and Conflict.

Finally, we included period effects in the models of Table 6 in order to test whether the effects of intentions on contributions changed over time, a dynamic that Figure 5 suggests. In general, we did find significantly decreasing contributions [38]. However, the model with the interaction effects demonstrates that the period effects hardly differed between the Harmony and the Conflict condition. Likelihood ratio tests revealed that including interaction terms between the period-variable and the own intentions, the higher intention of the group and the lower intention did not increase model fit significantly. This demonstrates that the effects that the intentions had on contributions did not change significantly over time.

In a nutshell, analyses of the standardized messages revealed that subjects tended to contribute more points when their group members stated that they would contribute more. We found this pattern in both conditions, concluding that subjects did prefer fair payoff distributions amongst the members of their group. However, subjects adjusted their contributions mainly in accordance with the group member that stated the lower intention and, actually, contributed on average even fewer points than stated by the lower intention. This might explain why having the opportunity to transmit standardized statements did not increase contributions substantially.

#### Analysis of the short messages

The analyses of the previous section demonstrated that the statement of the intentions did not increase contributions significantly, failing to support the claim that the communication of intentions is the core process that explains why communication increases engagement in intergroup conflict. Thus, it remains an open question why intragroup communication in terms of short-message communication increased contributions and fueled intergroup conflict. In order to explore which processes might explain this effect, we analyzed the content of the short messages.

Obviously, short messages can contain a multitude of different pieces of information and arguments that might motivate individual contributions. In order to explore the content of the messages and whether receiving messages with a specific content affected subjects’ contributions, we asked three coders to independently from each other evaluate each of the 480 short messages that subjects had transmitted. This method is becoming a standard approach to study the content of communication in economic experiments [19], [20], [39], [40]. We created a list of mechanisms that existing contributions have proposed to explain why communication motivates higher contributions [20], [37], [41], [42]. Next, we formulated for each potential mechanism a coding question that asked whether the content of a message indicated that the mechanism might play a role (coded as 1) or not (coded as 0). Table 7 lists the mechanisms, and the respective coding tasks and provides for each mechanism an example of a message where all three coders answered the coding question with “yes”. It turned out that this method captured the content of most messages. In total, for 76.46 percent of all messages at least one of the coding questions was answered with “yes”. 53 messages from the Harmony treatment and 60 messages from the Conflict treatment were always coded with “no”. In Table 7, we also report the Cronbach’s Alpha for each coding question, which demonstrates that the inter-coder reliability was high.

**Table 7:** Aspects of communication.

Aspect	Coder instruction(short form)	Example of shortmessage (translated)	Cronbach’sAlpha
Statement of intentions	Does the sender of the messageexplicitly state howmany points he/she isgoing to contribute?	“I will contribute 10 points.”	.709
Sending of norms	Does the sender of the message statehow much he/she wantsthe others to contribute?	“I will contribute 10 points again.I hope you’ll do the same.”	.583
Persuasion	Does the sender seekto convince the others,using an argument?	“The best is to contribute everything,also in order to seehow the others will react”	.827
Perceived groupcompetition	Does the messagemention the other group?	“Nice. Our strategy begins to work out.Keep it up, then Greenwill have less.”	.960
Loss aversion	Does the sender raise concerns thathigh contributions are theonly way to prevent losses?	“We have to contribute the maximum.Otherwise we will get negative points”	.810
Verbalpunishment	Does sender complain aboutlow contributions of others?	“Stealing 5 cents from us, person whocontributed 7 :P I will have to buy bread.”	.798
Positive reinforcement	Does the sender supportthe others in their previous behavior?	“Well done team :)Go ahead with 10 points!”	.662
Communication createsgroup identification	Does sender refer to agroup identity/team work?	“Team Blue rules!”(not translated)	.955

**Figure 6:**
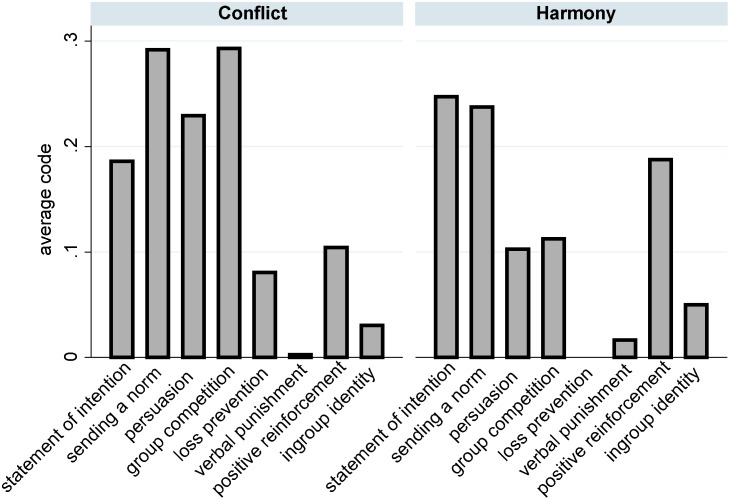
Content of the short messages in Conflict and Harmony.

For each message and each potential mechanism we calculated the average rating across the three coders, arriving at outcome measures that vary between zero and one. A value of zero is adopted if none of the coders thought that the content of the message indicates that the respective mechanism played a role. The outcome measures adopt the value 1 if all three coders consistently answered the coding question with “yes”. Figure 6 provides an overview of the content of the messages, showing the average of the outcome measures over all messages. The bars depict the share of the messages that indicated that the respective mechanisms might have played a role, weighted by the consistency of the coder ratings. The sum of the bars can exceed the value of one because messages can contain aspects of multiple mechanisms. For instance, the message “I will contribute 10 again! I hope you too” was coded by all three coders as a message that stated an intention and formulated a norm. The figure shows that messages were mainly used to inform subgroup members about one’s intentions and to articulate expectations about how much everybody should contribute. In the Harmony condition, messages frequently contained persuasive arguments and references to the other group. Positive reinforcement was used more often in the Harmony condition than in Conflict. What is more, Figure 6 shows that subjects hardly referred to a common subgroup identity and hardly used verbal punishment. This fails to support that these mechanisms caused the high contributions in the condition with short-message communication.

Finally, we tested whether subjects’ contribution decisions were affected by the content of the short messages that their subgroup members had transmitted. Therefore, we calculated for each contribution decision and each potential mechanism the average rating of the two messages that the two subgroup members had transmitted before the decision. These measures were entered as independent variables in random-intercept models with contributions as dependent variable (see Table 8). Like in the previous analyses, we controlled for the nestedness of decisions in subjects. Model 1 replicates the finding that subjects contributed significantly more points in the Harmony condition (see also the full model in Table 5). In Model 2 we added the measures of the messages’ content, which did not increase model fir significantly according to the likelihood-ratio-test (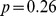). Positive (negative) effects indicate that subjects contributed more (fewer) points after having received messages with the respective content. The table shows that only messages that contained positive reinforcement affected contributions significantly. To put it more precisely, contributions were significantly higher when subjects received messages that evaluated their past contributions positively. This statistical effect turned out to be robust also when all insignificant variables from Model 2 are excluded (see Model 3). A likelihood-ratio-test showed that the fit of Model 3 was significantly better than the fit of Model 1 (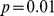). We also tested whether the statistical effect of receiving positive reinforcement might have been caused by the relationship with the opposite causal order because it appears plausible that subjects who contribute many points also tend to receive positively reinforcing messages more frequently. In order to statistically control for this possibility, we included in Model 4 the lagged dependent variable. Obviously, when a lagged dependent variable is included, large parts of the subject-level variance are captured by the effect of the lagged variable. Presumably, this was the reason why MLwiN’s IGLS algorithm failed to provide estimates for the subject-level variation. We also estimated models based on MLwiN’s IGLS, and MCMC, and Stata’s restricted maximum likelihood estimation, which did provide estimates. All approaches lead to very similar results, both in the random and in the fixed part of the model. Furthermore, OLS models also resulted in very similar estimates. In Table 8, we report MCMC estimates for Model 4 [43]. Strikingly, the effect of receiving messages that contain positive reinforcement remained significant, demonstrating that subjects who received such a message increased their contributions.

**Table 8:** Effects of message content.

	Dependent variable: Contribution			
	Model 1	Model 2	Model 3	Model 4
*Fixed part*				
Constant	8.93***	8.78***	8.81***	8.05***
	(0.16)	(0.22)	(0.15)	(0.43)
Harmony	0.71***	0.62***	0.62***	0.60***
condition	(0.22)	(0.23)	(0.21)	(0.19)
Statement of		0.03		
intention		(0.41)		
Sending a norm		−0.16		
		(0.41)		
Persuasion		−0.16		
		(0.42)		
Group		0.17		
competition		(0.34)		
Loss prevention		0.39		
		(0.76)		
Verbal		1.45		
punishment		(1.42)		
Positive		1.13**	1.11***	0.74*
reinforcement		(0.45)	(0.42)	(0.41)
Group identity		0.77		
		(0.65)		
Contribution			0.10**	
previous period			(0.05)	
*Random part*				
between subject	0.24	0.21	0.19	0.04
variance	(0.12)	(0.11)	(0.11)	(0.06)
Residual	3.39	3.34	3.37	3.27
variance	(0.23)	(0.23)	(0.23)	(0.23)
−2 loglikelihood	1973.73	1963.62	1966.84	1935.90
Number of subjects	48	48	48	48
Number of decisions	480	480	480	432

Standard errors in parentheses.

***significantly different from 0 at the 1 percent level (twosided Wald-test).

**significantly different from 0 at the 5 percent level (twosided Wald-test).

*significantly different from 0 at the 10 percent level (twosided Wald-test).

In a nutshell, we found that subjects who received positive feedback contributed significantly more points. On the one hand, this suggests that positive reinforcement might explain the effect of communication on contributions. On the other hand, Figure 6 showed that subjects used positive reinforcement relatively rarely in their messages. What is more, the statistical effect of receiving positive feedback on contributions (see Table 8) is not large and fails to explain the big difference in contributions between the conditions without communication and communication in terms of short messages (see Table 5). Indicating that positive reinforcement fails to explain the strong effects of communication in terms of short messages, the intercepts of Models 1 and 3 in Table 8 do not differ significantly.

Strikingly, our explorative analysis did not provide evidence in support of the remaining mechanisms. Even though subjects frequently stated intentions, formulated norms, tried to persuade, and referred to the other group, contribution decisions turned out to be unaffected by these messages. However, this does not rule out that some of the mechanisms played a considerable role. For instance, it has been argued that group identities form unconsciously [44], suggesting that communication in terms of short messages might have created a group identity but group members did not articulate this in their messages. Nevertheless, it is questionable whether it is possible that mechanisms like persuasion, verbal punishment, and social norms can motivate contributions without being manifest in the content of the short messages. Another explanation for the limited effects could be that subjects were not given identifiers that would have allowed them to comment on the behavior of a specific member of their group. Future studies should explore whether more sophisticated communication is found when participants can address individual group members easier than in our design.

## Summary and Discussion

What have our studies taught us about social preferences and intergroup conflict? First, Experiment 1 demonstrated that intergroup settings do not necessarily generate negative social preferences towards members of the outgroup and do not always motivate individuals to harm outgroup members. This result is consistent with findings from earlier game theoretic research [6], [8], but challenges a core assumption of social psychology’s minimal-group paradigm [10], [13], which claims that even a so called “minimal” (random) categorization of individuals suffices to create the striving for increased differences between groups. Inspired by this claim, we randomly assigned subjects to subgroups. However, in addition we imposed that contributions to the public good of one’s group harmed outgroup members, creating more than “minimal” group boundaries. Nevertheless, we did not find support for negative social preferences towards outgroup members or, in other words, outgroup hate.

To be sure, the lack of support for outgroup hate in our experiments does not exclude that outgroup hate might play a critical role in other intergroup settings and can motivate contributions to conflict. However, our results show that individuals may engage in intergroup conflict without feelings of outgroup hate. This is an important finding because it can not be explained with classical theories of intergroup relations [10], [13], [14]. These approaches to conflict focus on the conditions under which individuals seek to harm outgroup members and neglect the fact that conflict might be a mere byproduct of intragroup processes that do not involve outgroup hate. Similarly, our results suggest that intervention programs that seek to establish more harmonious intergroup relations by reducing outgroup hate, such as the famous “common ingroup identity model” [45], may have limited effects when conflicts are not the result of outgroup hate.

Likewise, our results do not exclude that a minimal group categorization might create ingroup love in the sense that subjects have positive social preferences towards members of the ingroup, an effect which found support in recent game theoretic experiments [12]. Further support for the notion that mere categorization can entail an ingroup bias was found in the first experiment. In the Harmony condition, subjects contributed more when they expected that their group members contribute many points. They did not respond to expected contributions by outgroup members, however. Future experimental research is needed to identify conditions under which group categorization creates group biases, as well as positive and negative social preferences towards in and outgroups.

Our second experiment provided new support for the striking claim that populations can suffer from intergroup conflict even though individuals do not seek to harm outgroup members because processes that act within subgroups motivate high contributions and fuel intergroup conflicts [6]. In agreement with earlier social psychological research [7], [8], we found that subjects contributed on average more than 90 percent of their endowment when they could transmit short messages with their fellow group members.

Extending earlier studies, we sought to identify the mechanism that underlies this communication effect, trying to open the black box of communication research that has been criticized earlier [5]. In particular, we tested whether within-subgroup communication increases contributions to intergroup conflict because individuals inform each other about their intentions and tend to stick to these intentions, a mechanism that has recently been included in rigorous models of strategic decision making [21], [23], [24]. Our results do not provide support for this reasoning. In the experimental conditions where subjects informed their group members about their intentions, contributions were not significantly higher than in the conditions without communication and substantially smaller than in the conditions where subjects transmitted short messages. We did find that contributions reflected at least to some degree the intentions that subjects stated and that contributions were influenced by the stated intentions of fellow group members. However, subjects contributed fewer points than they stated in their intention messages, sapping the proposed effect of intention communication. In the experiment, statements of intentions were cheap talk and contributing fewer points than stated could not lead to any kind of punishment. It would, therefore, be interesting to investigate contributions when stating intentions implies costs in such a way that stating the intention to contribute credibly signals cooperativeness.

In sum, it remains an open question why communication in terms of short messages increased contributions and fueled intergroup conflict. Our explorative analyses of the content of messages provided some support for the argument that group members tend to praise high contributions of group members and, thus, provide selective social incentives to contribute. However, statistical effects were rather small, suggesting that this mechanism accounts only for parts of the communication effect. Nevertheless, future research might take this finding as a starting point and conduct an experiment where subjects can send standardized feedback messages to their group members.

Identifying the mechanisms that cause communication effects is an intricate problem, because multiple mechanisms might interact and add up to the overall effect of communication. Nevertheless, the apparent contradiction between the theoretical insight that contributions to intergroup conflict are neither individually rational nor collectively efficient, on the one hand, and the high contributions that experimenters observed when subjects could communicate, on the other hand, makes it an important endeavor for future research.

## Supporting Information

File S1 **Screenshots of the experimental instructions and the main stages of the experiment.**
(PDF)
