# Supplementary material for: Do Intergroup Conflicts Necessarily Result from Outgroup Hate?
Source: PLoS One. 2014 Jun 4;9(6):e97848. doi: 10.1371/journal.pone.0097848 (PMC4045668; doi:10.1371/journal.pone.0097848)
Supplement: File S1 — Screenshots of the experimental instructions and the main stages of the experiment. (PDF) [file pone.0097848.s001.pdf]

"The opposite of love is not hate; it's indifference." Do intergroup conflicts necessarily result from outgroup hate?

Michael Mäs[1,*], Jacob Dijkstra[2]
1 ETH Zurich, Chair of Sociology, in particular of Modeling and Simulation, Zürich, Switzerland
2 Department of Sociology / ICS, University of Groningen, Groningen, The Netherlands
∗ E-mail: Corresponding mmaes@ethz.ch

Supporting Information

The instructions of the first and the second experiment were very similar. Therefore, we focus here on the instruction of the first experiment. In both experiments, subjects read all instructions on the computer screens. Figure S1 shows a screen shot of the first part of the instructions, which focussed on the intragroup public good problem. This screen was shown to all subjects at the beginning of the experiment, irrespective of the experimental condition. The information that is shown in Figure S1 was also included in the oral instructions at the very beginning of the experiment.

Subsequently, subjects answered a set of questions about the instructions and read the correct answers to these questions. On the next screen, subjects were informed about the interdependence between the their ingroup and the outgroup. For instance, those subjects who were assigned to the "first Harmony, then Conflict treatment" saw the screen that is shown in Figure S2. The same screen was presented to those subjects who entered the Harmony condition in the "first Conflict, then Harmony treatment" at the beginning of the eleventh period. In order to make sure that subjects were not able to identify the members of their group and the other group, we used the same group labels for all subjects (own group was always labeled "BLUE"; other group was always labeled "GREEN"). Figure S3 shows how subjects entered their contribution decisions. The summary of the instructions that was shown at this stage (see bottom of screen shown in Figure S3) was presented at all stages of the experiment. The exact instructions for the guessing, however, were only shown while subjects entered their guesses.

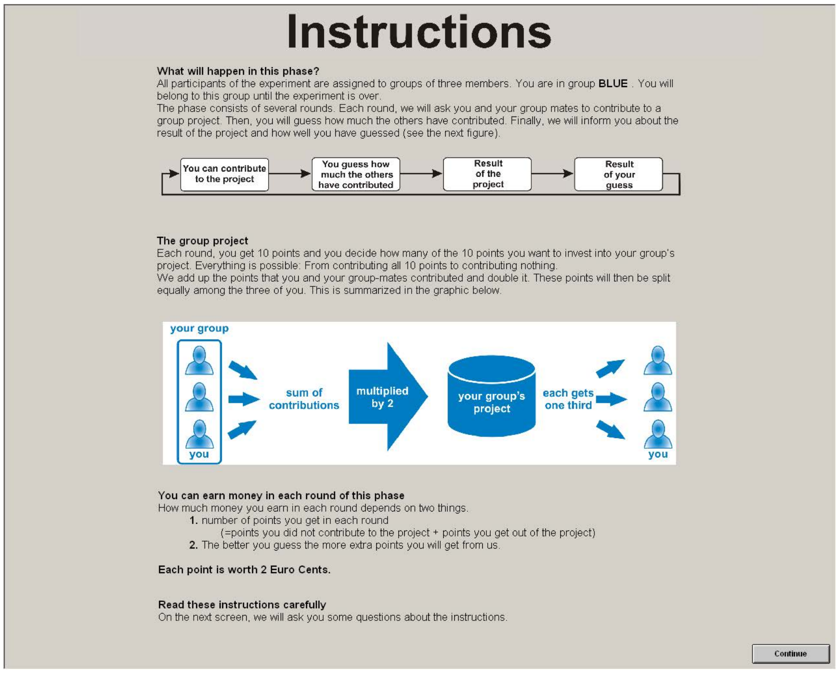

Figure S1. Instructions of the intragroup public-good setting

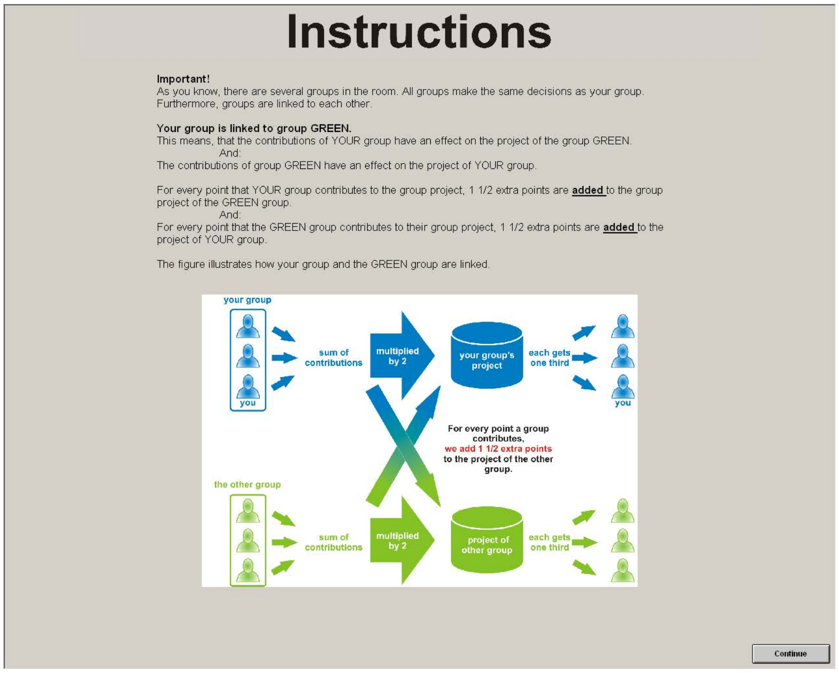

Figure S2. Instructions of the intergroup setting (screen shot from the Harmony condition)

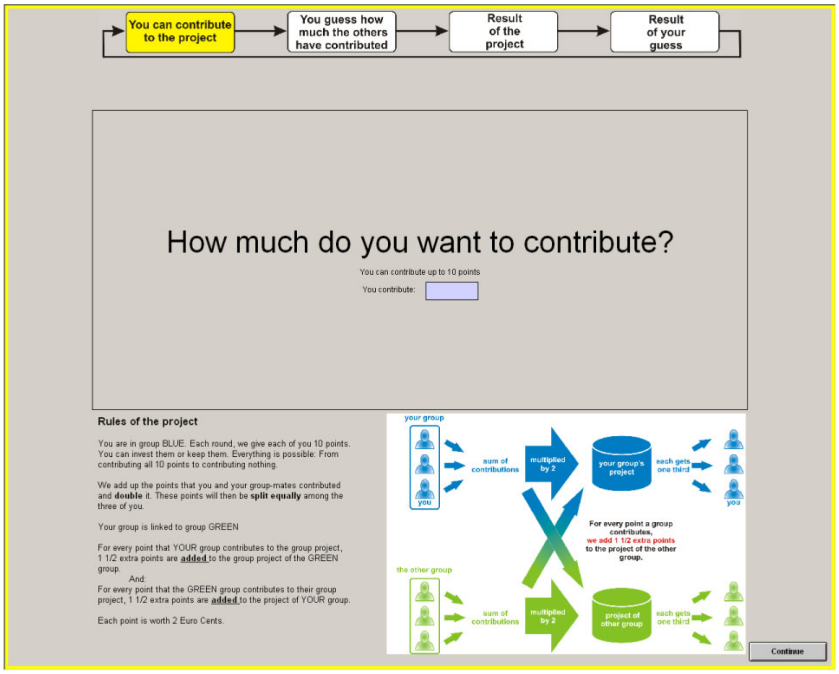

Figure S3. Contribution stage of the experiment (screen shot from the Harmony condition)
